# Epigenetically Controlled *ZEB2* Expression Promotes the Cytotoxic Potential of CMV‐Specific CD8^+^ T Cells

**DOI:** 10.1002/eji.70084

**Published:** 2025-11-06

**Authors:** Varun Sasidharan Nair, Zheng Yu, Hosein Ahmadi, Agnes Bonifacius, Beate Pietzsch, Dirk H. Busch, Luka Cicin‐Sain, Fabian Müller, Kilian Schober, Britta Eiz‐Vesper, Stefan Floess, Jochen Huehn

**Affiliations:** ^1^ Department Experimental Immunology Helmholtz Centre for Infection Research Braunschweig Germany; ^2^ Institute of Transfusion Medicine and Transplant Engineering Hannover Medical School Hannover Germany; ^3^ German Center for Infection Research (DZIF) Thematic Translation Unit‐Immunocompromised Host (TTU‐IICH), Partner Site Hannover‐ Braunschweig Germany; ^4^ nextGENERATION Medical Scientist Program, Dean's Office For Academic Career Development Hannover Medical School Hannover Germany; ^5^ Institute For Medical Microbiology, Immunology and Hygiene Technical University Munich (TUM) Munich Germany; ^6^ German Center for Infection Research (DZIF) Thematic Translation Unit‐Immunocompromised Host (TTU‐IICH), Partner Site Munich Germany; ^7^ Department of Viral Immunology Helmholtz Centre for Infection Research Braunschweig Germany; ^8^ Center For Individualised Infection Medicine (CiiM) a Joint Venture Between the Hannover Medical School and the Helmholtz Centre For Infection Research Hannover Germany; ^9^ Cluster of Excellence RESIST (EXC 2155) Hannover Medical School Hannover Germany; ^10^ Integrative Cellular Biology and Bioinformatics Saarland University Saarbrücken Germany; ^11^ Mikrobiologisches Institut – Klinische Mikrobiologie Immunologie Und Hygiene, Universitätsklinikum Erlangen Und Friedrich‐Alexander‐Universität (FAU) Erlangen‐Nürnberg Erlangen Germany; ^12^ FAU Profile Center Immunomedicine Friedrich‐Alexander‐Universität (FAU) Erlangen‐Nürnberg Erlangen Germany

**Keywords:** CD8^+^ T cells, cell–cell adhesion, cytotoxicity, epigenetics

## Abstract

Zinc finger E‐box binding protein 2 (ZEB2) is a key factor in the differentiation of naïve CD8^+^ T cells into effector and memory T cells. However, the precise regulatory role of ZEB2 in cytotoxic CD8^+^ T cells remains unknown. Our recent DNA methylation analysis of cytomegalovirus (CMV)‐specific human CD8^+^ T cells revealed two differentially methylated regions (DMRs) within the *ZEB2* locus. In the present study, we show that these *ZEB2* DMRs undergo pronounced demethylation during T cell differentiation. In particular, terminally differentiated CD8^+^ T cells and cytotoxic CD4^+^ T cells show an almost complete demethylation. Demethylation of the *ZEB2* DMRs correlates strongly with *ZEB2* expression in all T cell subsets. Furthermore, DNA methylation patterns remain stable during long‐term in vitro culture. *ZEB2* knockout in CD8^+^ effector T cells results in altered gene expression profiles, affecting genes related to cell–cell adhesion and impairing the cytotoxic capacity in CMV‐specific killing assays. Our data show that *ZEB2* expression contributes to the differentiation of naïve CD8^+^ T cells into effector and memory T cells and regulates the functional properties of virus‐specific cytotoxic CD8^+^ T cells.

## Graphical abstract

Differentially methylated regions (DMRs) within the *ZEB2* locus progressively demethylate during CD4^+^ and CD8^+^ T cell differentiation into cytotoxic CD4^+^ and CD8^+^ T_EMRA_ subsets, respectively. This epigenetic remodeling tightly correlates with elevated *ZEB2* expression, and *ZEB2* knockout (KO) impairs the effector and cytotoxic function of CD4^+^ and CD8^+^ T cells.

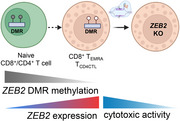

**Abbreviations**
CMV: cytomegalovirus
CTV: CellTrace Violet
DEG: differentially expressed gene
DMRs: differentially methylated regions
HLA: human leukocyte antigen
KO: knockout
pp65: phosphoprotein 65
RNP: ribonucleoprotein
T(CMV): CMV‐specific CD8^+^ T cells
T‐bet: T‐box expressing in T cells
T_CD4CTL_
: cytotoxic CD4^+^ T cells
T_CM_
: central memory T cells
T_EM_
: effector memory T cells
T_EMRA_
: effector memory cells re‐expressing CD45RA
Th: T‐helper
T_N_
: naive T cells
TPM: transcripts per million
T_SCM_
: stem‐cell‐like memory T cells
ZEB: Zinc finger E homeobox binding

## 1 Introduction

During viral infection, antigen‐specific naive CD8⁺ T cells get activated, expand clonally, and differentiate into effector cells that control viral replication and eliminate virus‐infected cells by direct killing and the release of cytokines and cytotoxic granules. Following pathogen clearance, the majority of effector CD8^+^ T cells undergo apoptosis, while a smaller number of long‐lived memory CD8^+^ T cells prevails, thereby ensuring long‐term immunity [1].

CD8^+^ T cell differentiation is orchestrated by complex transcriptional programmes [2] that are accompanied by extensive epigenetic modifications, including DNA methylation, histone modifications, and noncoding RNAs, which result in substantial changes in the transcriptional landscape of key regulatory genes, guiding the development and function of effector and memory T cell subsets [3, 4, 5]. Although groundbreaking genome‐wide epigenetic studies in human CD8^+^ T cells have shown that DNA methylation is the most important regulator for maintaining transcriptionally active and inactive chromatin structures in differentiated cells [6, 7, 8], the DNA methylation pattern of virus‐specific human CD8^+^ T cells remained elusive.

Recently, we performed a DNA methylation profiling study of cytomegalovirus (CMV)‐specific CD8^+^ T cells T(CMV) and identified several differentially methylated regions (DMRs) associated with transcription factors, including *ZEB2* [9]. ZEB2, along with its homolog ZEB1, is primarily known for its role in epithelial‐to‐mesenchymal transition, but has also been reported to modulate T cell differentiation [10]. In murine effector CD8^+^ T cells following lymphocytic choriomeningitis virus (LCMV) infection, Zeb2 is upregulated, and loss of *Zeb2* is associated with significant impairments in the development of antigen‐specific CD8^+^ T cells and effector/memory subsets [11]. Furthermore, as a potential downstream target of T‐bet, Zeb2 has been reported to coordinate with T‐bet to regulate the transcriptional network of effector T cells [11].

In the present study, we investigated the DNA methylation status of the recently identified *ZEB2* DMRs in human T cell subsets and observed an almost complete demethylation in terminally differentiated CD8^+^ T cells (T_EMRA_) and cytotoxic CD4^+^ T cells (T_CD4CTL_), which correlated with strong *ZEB2* expression. Knockout (KO) of *ZEB2* in CD8^+^ effector T cells resulted in impaired expression of genes related to cell–cell adhesion and also reduced their cytotoxic capacity. Collectively, these findings demonstrate that *ZEB2* expression in human effector CD8^+^ T cells is subject to epigenetic regulation and directly influences the cytotoxic capacity of virus‐specific CD8^+^ T cells.

## 2 Results and Discussion

### 2.1 ***ZEB2*** expression Strongly Correlates with Methylation Status of *ZEB2* DMRs in CD8^+^ T Cell Subsets

**FIGURE 1:**
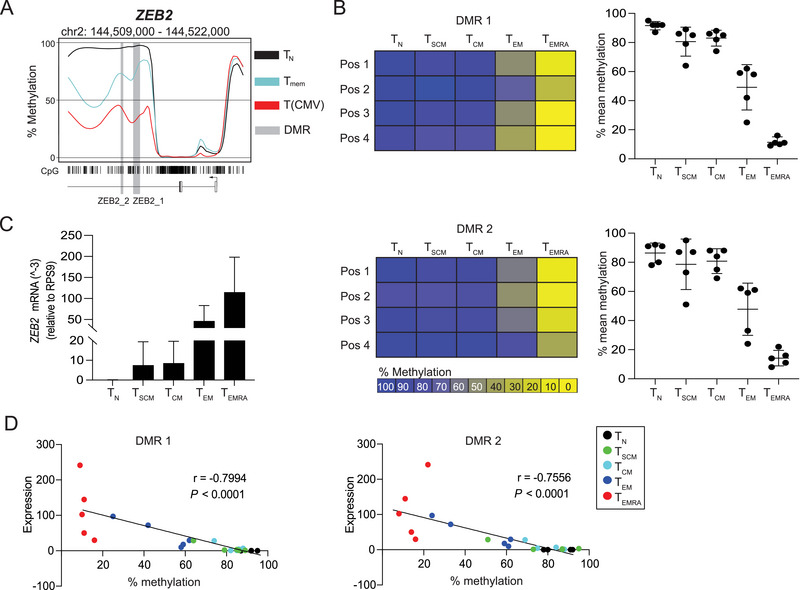
Methylation status of *ZEB2* DMRs correlates with *ZEB2* expression in CD8^+^ T cell subsets. CD8^+^ T subsets from CMV‐seropositive healthy donors were sorted by flow cytometry, and gDNA and RNA were isolated. Bisulfite‐converted gDNA was subjected to pyrosequencing of *ZEB2* DMRs, and RNA was used to quantify *ZEB2* expression. (A) *ZEB2* gene locus showing the position of DMR1 (size: 471 nt; position: 144,513,934–144,514,404) and DMR2 (size: 143 nt; position: 144,513,024–144,513,166) relative to elements of the gene; modified from Yu et al. [9]. (B) Methylation values of individual CpG motifs in *ZEB2* DMR1 (top) and *ZEB2* DMR2 (bottom) in indicated human CD8^+^ T cell subsets from one representative donor were plotted in a heat map (left), and mean methylation levels for all CpG motifs within the indicated DMRs from five independent donors (mean ± SD) are shown in the graphs (right). Methylation values of individual CpG motifs have been transformed into a color‐coded box, ranging from yellow (0%) to blue (100%). (C) Bar plots show *ZEB2* expression relative to the housekeeping gene *RPS9* in CD8^+^ T cells from CMV‐seropositive donors (*n* = 5). (D) Scatterplots show correlation of the mean methylation of *ZEB2* DMR1 (left) and DMR2 (right) with *ZEB2* expression in CD8^+^ T_N_ (black), T_SCM_ (green), T_CM_ (cyan), T_EM_ (blue), and T_EMRA_ (red) cells (*n* = 5). Linear regression analysis was performed to determine the relationship.

In a previous DNA methylation profiling study, two DMRs were found within the *ZEB2* locus (Figure 1A), showing a pronounced demethylation in T(CMV) [9]. To study the role of ZEB2 in T(CMV), we first investigated the DNA methylation status of the two *ZEB2* DMRs in naive (T_N_), stem cell‐like memory (T_SCM_), central memory (T_CM_), effector memory (T_EM_) and effector memory cells re‐expressing CD45RA (T_EMRA_) CD8^+^ T cell subsets from CMV‐seropositive donors (Figure S1A). For both *ZEB2* DMRs, T_EM_ and T_EMRA_ cells showed a partial and pronounced demethylation, respectively, whereas T_N_, T_SCM_, and T_CM_ cells were extensively methylated (Figure 1B). This DNA methylation pattern is in accordance with the reported predominance of T_EM_ and T_EMRA_ phenotypes among T(CMV) [12, 13] and suggests that ZEB2 may be involved in both differentiation and effector function of antigen‐specific CD8^+^ T cells during CMV infection.

We then quantified *ZEB2* expression in the T cell subsets to correlate DNA methylation with gene expression. While T_N_ cells lacked *ZEB2* expression, a weak expression was observed in both T_SCM_ and T_CM_ cells, and T_EM_ and T_EMRA_ cells showed an intermediate and high expression, respectively (Figure 1C), in line with previous findings suggesting a contribution of ZEB2 to terminal T cell differentiation [11, 14]. Correlation analysis revealed a significant inverse correlation between mean *ZEB2* expression and mean DNA methylation levels of both *ZEB2* DMRs across all CD8^+^ T cell subsets (Figure 1D). Similar findings were observed for cells from CMV‐seronegative donors (Figure S2). Taken together, our data indicate that the two *ZEB2* DMRs were progressively demethylated during CD8^+^ T cell differentiation, enabling the expression of the transcription factor ZEB2 in T_EM_ and T_EMRA_ cells.

### 2.2 CD8^+^ T_N_ and T_EMRA_ Cells Maintain Their *ZEB2* DMRs Methylation Patterns During Long‐Term In Vitro Culture

Next, we investigated the epigenetic stability of the *ZEB2* DMRs in fully methylated CD8^+^ T_N_ cells and highly demethylated T_EMRA_ cells by long‐term in vitro culture for up to 30 days with regular assessment of the *ZEB2* DMRs methylation patterns. Both T_N_ and T_EMRA_ cells showed very stable DNA methylation patterns and remained methylated and strongly demethylated, respectively, at both *ZEB2* DMRs (Figure S3). Thus, although a gradual, genome‐wide loss of DNA methylation occurs with T cell memory differentiation and correlates with enhanced cell proliferation [15, 16], our findings argue against a proliferation‐driven demethylation process and rather suggest a specific role for these DMRs in regulating *ZEB2* expression.

### 2.3 Methylation Patterns of *ZEB2* DMRs Correlate with *ZEB2* Expression in CD4^+^ T Cell Subsets

A subpopulation of CD4^+^ T cells, CD57^+^CD27^–^CD28^–^CD244^+^ cytotoxic CD4^+^ T cells (T_CD4CTL_), is preferentially found in CMV‐seropositive individuals, able to control CMV expansion, and characterised by *ZEB2* expression [17]. We here sorted CD4^+^ T cell subsets, including T_N_, T_CM_, T_EM_, and T_CD4CTL_, from CMV‐seropositive donors (Figure S1B) and assessed the methylation pattern of *ZEB2* DMRs and *ZEB2* expression. Both *ZEB2* DMRs showed comparable DNA methylation patterns, with T_CD4CTL_ cells displaying an almost complete demethylation (Figure S4A). CD4^+^ T_EM_ cells were only weakly demethylated, whereas both T_N_ and T_CM_ cells were largely methylated (Figure S4A). Quantification of *ZEB2* expression revealed the highest expression levels in T_CD4CTL_ followed by T_EM_ cells, whereas rather low expression levels were found in T_N_ and T_CM_ cells (Figure S4B), resulting in a significant inverse correlation between mean *ZEB2* expression and mean DNA methylation levels of both *ZEB2* DMRs across all CD4^+^ T cell subsets (Figure S4C). Our observation that T_CD4CTL_ expressed the highest levels of *ZEB2* is in line with a recent analysis of CD4^+^ T cell subsets from autoimmune disease patients, which revealed that ZEB2 regulates the cytotoxic phenotype of age‐associated CD4^+^ T cells [18], further supporting the hypothesis that ZEB2 plays a functional role in cytotoxic T cells.

### 2.4 *ZEB2* KO in CD8^+^ T Cells Affects Expression of Genes Involved in Cell–Cell Interaction

**FIGURE 2:**
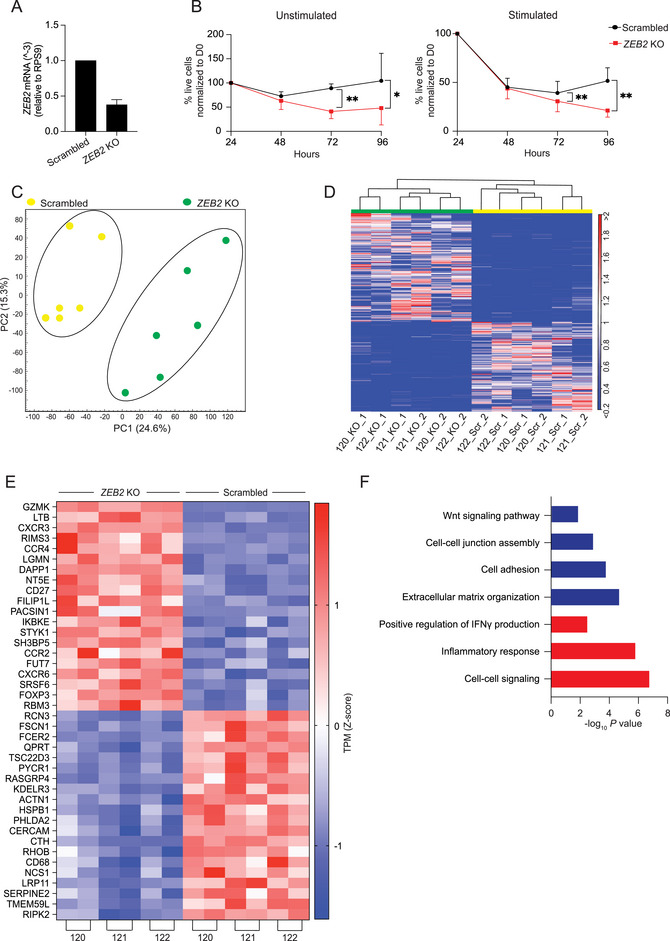
*ZEB2* deletion profoundly alters transcriptional profile of CD8^+^ T cells. CD8^+^ T_EM_ and T_EMRA_ cells were isolated from 3 CMV‐seronegative donors. Sorted cells were stimulated for 5 days, subsequently transfected with RNPs targeting *ZEB2* or scrambled controls, and samples were subjected to RNAseq. (A) Two days after nucleofection, the KO efficacy was assessed by qRT‐PCR. The bar plot shows *ZEB2* relative to *RPS9* expression in KO and scrambled control samples. (B) Line plots show viability of transfected cells at the indicated time points after nucleofection, determined by flow cytometry. (C) Plot showing top two principal components distinguishing the transcriptional profiles of *ZEB2* KO (green) and scrambled control samples (yellow). (D) Euclidean distance analysis and unsupervised hierarchical clustering of top 5000 DEGs between *ZEB2* KO and scrambled control CD8^+^ T cells. Color represents expression level, ranging from deep blue (<0.2) to deep red (>2). (E) Unsupervised hierarchical clustering of top 40 DEGs (20 up‐ and 20 downregulated) from pairwise analysis between *ZEB2* KO and scrambled controls with FC>1.5 and *p*‐value <0.05 cut‐off. Expression level of each gene was plotted by TPM values, and color coding is based on *z*‐score, ranging from −1.5 (blue) to +1.5 (red). (F) Up‐ and downregulated genes (with fold change >1.5 and *p*‐value < 0.05) were subjected to gene ontology (GO) analysis. Bar plot shows −log_10_ of the *p*‐value of indicated GO terms. Blue bar, enriched GO terms regulated by downregulated genes in *ZEB2* KO; red bar, enriched GO terms regulated by upregulated genes in *ZEB2* KO.

To identify downstream targets of ZEB2, we investigated the impact of *ZEB2* KO on the transcriptome of CD8^+^ effector T cells. For this, we sorted effector CD8^+^ T cells, including both T_EM_ and T_EMRA_ cells, which showed the highest *ZEB2* expression levels, and performed CRISPR‐Cas9‐mediated *ZEB2* KO. Quantification of *ZEB2* mRNA expression levels revealed a KO efficiency of approximately 60% (Figure 2A), similar to what was found in a previous study [11]. Strikingly, we found that *ZEB2* KO resulted in a significant reduction in viability at 72 h postgene deletion, regardless of stimulation condition (Figure 2B; Figure S5A), suggesting that ZEB2 is not only critical for differentiation and functional specialization, but also for CD8^+^ T cell survival. We therefore decided to generate transcriptomes of *ZEB2* KO and scrambled control CD8^+^ effector T cells on day 2 after CRISPR‐Cas9‐mediated genome editing, before strong differences in viability were observed.

To delineate the downstream targets of ZEB2, the RNAseq data of *ZEB2* KO effector CD8^+^ T cells and scrambled control CD8^+^ effector T cells were compared. Principal component analysis revealed a clear separation between *ZEB2* KO and scrambled control CD8^+^ T cells from independent donors (Figure 2C). This relationship was further demonstrated by Euclidean distance analysis and hierarchical clustering of the top 5000 differentially expressed genes (DEGs) (Figure 2D). We then classified genes that were differentially expressed (≥1.5‐fold change with a *p*‐value ≤ 0.05) (Table S1), and evaluated the top 20 up‐ and downregulated genes (Figure 2E). *GZMK* was among the top genes that were upregulated upon *ZEB2* KO, and assessing its expression in ex vivo isolated CD8^+^ T cell subsets revealed that T_EMRA_ cells, which showed the highest *ZEB2* expression, displayed the lowest *GZMK* expression among antigen‐experienced T cell subsets, particularly upon stimulation (Figure S5B). These observations suggest that ZEB2 prevents the generation of GZMK^+^CD8^+^ T cells, which have recently been shown to exhibit minimal cytotoxicity activity. Instead, they produce high levels of cytokines and efficiently activate the complement cascade, thereby amplifying chronic inflammation in tissues [19, 20, 21]. Notably, *ZEB2* KO in T_CD4CTL_ also results in the increased and decreased expression of genes coding for effector molecules (Figure S5C), indicating that ZEB2 exerts both positive and negative effects on gene expression in CD4^+^ and CD8^+^ T cells. Global analysis of all DEGs upon *ZEB2* KO in CD8^+^ T cells revealed that, in the absence of *ZEB2*, there was a pronounced downregulation of genes associated with cell adhesion and cell–cell junction assembly (Figure 2F; Table S1). Interestingly, there was no significant change in the majority of genes involved in CD8^+^ T cell cytotoxicity, whereas genes involved in cellular signaling, inflammatory response, and interferon‐gamma production even tended to be upregulated upon *ZEB2* loss (Figure 2F; Table S1). These data suggest that *ZEB2* KO did not adversely affect the canonical effector network of CD8^+^ T cells, but instead resulted in downregulation of genes associated with extracellular matrix organization, cell adhesion, cell–cell junction assembly, and the Wnt signaling pathway (Figure 2F; Table S1). Mapping possible protein–protein associations of significantly downregulated genes upon *ZEB2* loss revealed a robust interactive network that was highly enriched for genes related to cell adhesion (Figure S5D). Together, these data suggest that *ZEB2* KO CD8^+^ T cells have defects in cell targeting, which may account for an impairment in cytotoxicity.

### 2.5 *ZEB2* Deletion Reduces the Cytotoxic Capacity of CMV‐Specific CD8^+^ T Cells

**FIGURE 3:**
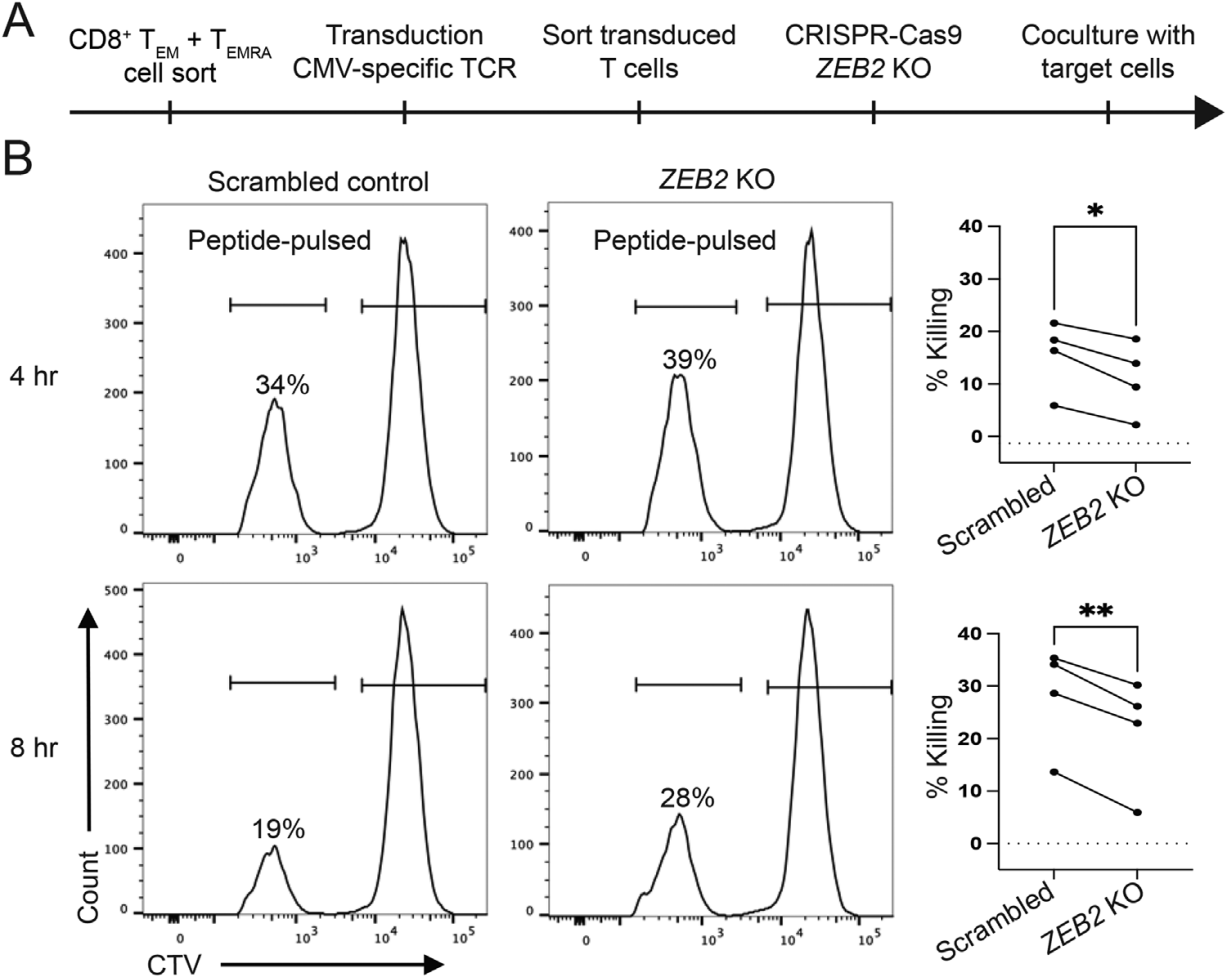
*ZEB2* deletion reduces cytotoxic capacity of CMV‐specific CD8^+^ T cells. CD8^+^ T_EM_ and T_EMRA_ cells isolated from CMV‐seronegative HLA‐A*02:01 negative donors were transduced with mTCR 5‐2 and subsequently transfected with RNPs targeting *ZEB2* or scrambled controls. HLA‐A*02:01‐transduced K562 cells, labelled with a low concentration of CTV, were pulsed with CMVpp65 peptide NLVPMVATV and mixed in a 1:1 ratio with nonpulsed HLA‐A*02:01‐transduced K562 cells, labelled with a high concentration of CTV. *ZEB2* KO and scrambled control CD8^+^ T cells were then co‐cultured with target K562 cells at a 1:1 E:T ratio and harvested after 4 and 8 h. Cytotoxicity was determined via the reduced frequency of CMVpp65 peptide‐pulsed target K562 cells in co‐cultures. (A) Scheme shows workflow of cytotoxicity assay. (B) Representative histograms (left) show frequency of CMVpp65 peptide‐pulsed K562 cells co‐cultured with scrambled control or *ZEB2* KO CD8^+^ T cells at indicated time points. Graphs (right) show cytotoxicity of indicated CD8^+^ T cells from four independent experiments.

Given that *ZEB2* deficiency results in impaired cell–cell interactions, we next investigated the role of ZEB2 in CD8^+^ T cell cytotoxicity using an in vitro cytotoxicity assay in which *ZEB2*‐deficient and control CD8^+^ T cells (both transduced with the CMV‐specific mTCR 5‐2) were co‐cultured with CMVpp65‐pulsed K562 target cells (Figure 3A). Effector cell cytotoxicity of *ZEB2* KO and control CD8^+^ T cells was determined at specific time points by comparing the frequency of CMVpp65‐pulsed (CTV^low^) and nonpulsed (CTV^high^) K562 cells (Figure S5C). Already after 4 h of co‐culture, *ZEB2* KO T cells showed a significantly reduced ability to kill CTV^low^ target cells when compared with the scrambled control CD8^+^ T cells, and this effect was even more pronounced after 8 h (Figure 3B). It is tempting to speculate that *ZEB2* deletion impairs the formation of immune synapses between the CMV‐specific CD8^+^ T cells and their target cells, thereby reducing their cytotoxic capacity [22]. However, the pathway analysis (Figure S5B) did not reveal any impairment in cytotoxicity‐related signaling upon the loss of ZEB2 in CD8^+^ effector T cells, suggesting that ZEB2 may be essential for CD8^+^ T cells to exert optimal effector function through cellular adhesion and Wnt signaling, which are critical for effective targeting.

### 2.6 Data Limitations and Perspectives

While our study provides valuable insights into the epigenetic regulation of *ZEB2* expression and its role in the cytotoxic function of CD8^+^ T cells, some issues still need to be addressed. One major challenge was our inability to perform a gain‐of‐function experiment, as repeated attempts to overexpress *ZEB2* using viral systems were unsuccessful, likely due to the tight intrinsic regulation of *ZEB2* expression required for cellular homeostasis. Developing titratable or mRNA‐based systems for controlled *ZEB2* expression could enable future studies to investigate the influence of varying ZEB2 levels on T cell function without compromising cell viability. Although we identified two key DMRs associated with *ZEB2* expression, the precise mechanisms by which these regions regulate transcription are yet to be elucidated. Future studies could use CRISPR/dCas9‐based epigenome editing to alter the methylation status of these DMRs and directly evaluate the relationship between demethylation and *ZEB2* expression. Furthermore, extending our findings to other chronic viral infections, cancers, or autoimmune diseases could clarify whether ZEB2 plays a more broadly conserved role across immune contexts or is specifically relevant to CMV‐driven responses. Finally, given existing evidence of cooperative and hierarchical regulation in effector T cell differentiation, it would be valuable to investigate how ZEB2 interacts with other key transcription factors, such as T‐BET and EOMES.

## 3 Materials and Methods

### 3.1 Human Donors

Human samples in this study were obtained from healthy male donors at the Institute of Transfusion Medicine and Transplant Engineering, Hannover Medical School (MHH). This study was conducted in accordance with the tenets of the Declaration of Helsinki and approved by the Ethics Committee of the Hannover Medical School (MHH Ethics Committee votes 3639‐2017, 9001_BO‐K, 9255_BO_K_2020). Written informed consent was obtained from all donors. Pyrosequencing of the *ZEB2* DMRs and quantitative RT‐PCR were performed using cells from both CMV‐seropositive and CMV‐seronegative male donors, regardless of HLA type. Retroviral transduction, CRISPR/Cas9‐mediated *ZEB2* KO, and in vitro cytotoxicity assays were performed using cells from CMV‐seronegative, human leukocyte antigen‐A2‐negative (HLA‐A*02:01^–^) male donors. All other experiments were performed with cells from CMV‐seropositive male donors, irrespective of their HLA type. CMV serology and HLA‐typing were performed at the Institute of Transfusion Medicine and Transplant Engineering, MHH.

### 3.2 Cell Isolation

PBMCs were isolated from Leukocyte Reduction System cones using Ficoll‐based density gradient centrifugation (Lymphoprep; STEMCELL technologies) and SepMate tubes (STEMCELL technologies). FCS containing 10% DMSO was used to cryopreserve PBMCs. One day before the experiment, the cryopreserved PBMCs were thawed, rinsed with excess TexMACS medium (Miltenyi Biotec), and then incubated overnight at 37°C and 5% CO_2_ in TexMACS medium supplemented with 100 IU/mL recombinant human IL‐2 (Miltenyi Biotec).

### 3.3 Flow Cytometry

PBMCs were first stained with the viability dye (Zombie Aqua fixable viability kit, Biolegend, or LIVE/DEAD fixable near‐IR dead cell dye, ThermoFischer Scientific) at 4°C for 15 min in the dark. Afterwards, cells were washed with staining buffer (0.5% BSA in PBS) and pelleted by centrifugation at 400×*g* for 5 min at 4°C. Single‐cell suspensions were labelled with antibodies (Supporting Information Materials and Methods) for 30 min at 4°C, while chemokine receptor staining was performed at 37°C for 30 min. Cells were then washed and resuspended in staining buffer, followed by acquisition on an LSR‐II SORP (BD Biosciences) or sorting on a BD FACS ARIA‐II SORP (BD Biosciences). BD FACSDiva v8.0.1 and Flowjo v10 software (both BD Biosciences) were used for analysis.

### 3.4 Isolation of T Cell Subsets

For the isolation of CD8^+^ T cell subsets, PBMCs were either directly stained and subsequently sorted by flow cytometry or first enriched for CD8^+^ cells using anti‐CD8 MicroBeads and the automated magnetic activated cell sorting (autoMACS) system (both Miltenyi Biotec). The following T cell subsets were sorted: naïve (T_N_; CD3^+^CD8^+^CD45RA^+^CCR7^+^CD28^+^CD62L^+^CD95^−^), stem cell‐like memory (T_SCM_; CD3^+^CD8^+^CD45RA^+^CCR7^+^CD28^+^CD62L^+^CD95^+^), central memory (T_CM_; CD3^+^CD8^+^CD45RA^−^CCR7^+^CD62L^+^), effector memory (T_EM_; CD3^+^CD8^+^CD45RA^−^CCR7^−^CD62L^−^), and T_EMRA_ cells (CD3^+^CD8^+^CD45RA^+^CCR7^−^CD28^−^CD62L^−^). For the isolation of CD4^+^ T cell subsets, see Supporting Information Materials and Methods.

### 3.5 Pyrosequencing of *ZEB2* DMRs

Genomic DNA from ex vivo isolated CD8^+^ and CD4^+^ T cell subsets or from in vitro cultured T_N_ and T_EMRA_ cells was extracted using the DNeasy blood & tissue kits (Qiagen), followed by bisulfite conversion using the EZ DNA methylation‐lightning kit (Zymo Research). *ZEB2* DMRs were analyzed by pyrosequencing as previously described [9]. The following primers were used for amplification and subsequent sequencing:

*ZEB2* DMR1:

‘forward’ 5′‐GGGAATTGTTAGGATTTATTTGAATTGA‐3′

‘reverse’ 5′‐Bio‐AACTACCTCCTTCTCCTTTACTTTT‐3′

‘sequencing 1′ 5′‐AGAAATTTTTGGAAAGAAATA‐3′

‘sequencing 2′ 5′‐AGAATGGTATTTTATATAATTTT‐3′

*ZEB2* DMR2:

‘forward’ 5′‐GAGTGGAGGTGTTGGTAGTGATG‐3′

‘reverse’ 5′‐Bio‐TACCACACAACCTACCCCAATAC‐3′

‘sequencing 1′ 5′‐AGTGATGGTTAGAGGT‐3′

‘sequencing’ 2′ 5′‐TGAAGTTGGGATGGG ‐3′

### 3.6 Quantitative RT‐PCR of *ZEB2*

Total RNA from sorted CD8^+^ and CD4^+^ T cell subsets was isolated using the RNeasy Mini Kit (Qiagen), quantified spectrometrically (DeNovix), and transcribed into cDNA using the Transcriptor First Strand cDNA Synthesis Kit (Roche). SYBR green master mix (Roche), cDNA, and *ZEB2* primers (‘forward’ 5′‐CGCCACGAGAAGAATGAAGA‐3′; ‘reverse’ 5′‐GATTACCTGCTCCTTGGGTTAG‐3′) were used for mRNA quantification on a LightCycler 480 II (Roche). Data were analysed on a LightCycler 96 SW 1.1 (Roche).

### 3.7 CRISPR/Cas9‐Mediated *ZEB2* KO

For *ZEB2* KO, either T_CD4CTL_ or a mixture of sorted CD8^+^ effector T cells, including T_EM_ and T_EMRA_ cells, was used. Before *ZEB2* KO, T cells were stimulated with plate‐bound anti‐CD3 (1 µg/mL) and anti‐CD28 (0.5 µg/mL) antibodies for 5 days. After the culture, the cells were harvested and used for transfection with ribonucleoprotein (RNP) complexes (see Supplementary Materials and Methods).

### 3.8 RNAseq

Total RNA was extracted from *ZEB2* KO and scrambled control samples using the RNeasy Plus Mini Kit (Qiagen). The RNA quality was confirmed by RNA Integrity Number (RIN) >7 (Agilent 2100 Bioanalyzer; Agilent Technologies). The RNAseq library was generated from 500 ng total RNA using the Dynabeads mRNA DIRECT Micro Purification Kit (Thermo Fisher) for mRNA purification, followed by the NEBNext Ultra II Directional RNA Library Prep Kit (New England BioLabs). Libraries were sequenced on Illumina NovaSeq 6000 using the NovaSeq 6000 S1 Reagent Kit (100 cycles, paired‐end run) with an average of 5 × 10^7^ reads per sample. For processing and analysis, see Supplementary Materials and Methods.

### 3.9 Retroviral Transduction

Retroviral transduction of CD8^+^ T cells with a CMV‐specific TCR was performed as previously described [9] using the pMP71 plasmid‐encoded mTCR 5‐2, a HLA‐A*02:01‐restricted high‐avidity TCR that recognizes the CMVpp65‐derived peptide NLVPMVATV and contains a murine constant region (mTCR) for the isolation of successfully transduced cells [23] (see Supporting Information Materials and Methods).

### 3.10 In Vitro Cytotoxicity Assay

One day before the cytotoxicity assay, the target cells, HLA‐A*02:01‐transduced K562 cells [24], were labeled with CellTrace Violet (CTV) using the CellTrace Violet‐Cell Proliferation Kit (Invitrogen) at a final concentration of 0.1 µM (CTV^low^), and pulsed with 5 µg/mL CMVpp65 (NLVPMVATV) peptide (Proimmune) overnight. Unloaded cells labeled with CTV at a final concentration of 5 µM (CTV^high^) were used as a control for the calculation of the specific killing. After overnight incubation, peptide‐loaded HLA‐A*02:01^+^ K562 cells were harvested, washed to remove unbound peptides, and mixed with unloaded HLA‐A*02:01^+^ K562 control cells at a 1:1 ratio. Next, 50,000 mixed HLA‐A*02:01^+^ K562 cells were co‐cultured with either *ZEB2* KO or scrambled control CD8^+^mTCR 5‐2^+^ T_EM_/T_EMRA_ cells at different effector to target ratios (no T cells, 0.5:1, 1:1, and 2:1) in 96‐well U‐bottom plates as technical replicates. After different time points (4 and 8 h), cells were harvested, washed, and stained with LIVE/DEAD Fixable Near‐IR Dead Cell Dye (dilution factor 1:1000 in PBS) and then incubated at 4°C for 15 min in the dark. After live/dead cell staining, cells were further washed with PBS/BSA, stained for CD3 and CD8, and analyzed by flow cytometry. For each time point (4 and 8 h) and condition (*ZEB2* KO and scrambled control), the percentage of specific killing was calculated by subtracting the mean frequency of CTV^low^ cells from all technical replicates from the mean frequency of CTV^high^ cells from all technical replicates.

## 4 Statistical Analysis

Statistical analysis was performed using Prism software v 9.4.0 (GraphPad). The normal distribution of data points was checked using the Shapiro–Wilk normality test. A paired two‐tailed parametric *t*‐test was performed on samples that passed the normality test, and a nonparametric Wilcoxon matched‐pairs signed‐rank test was performed on samples that failed the normality test. All data are presented as mean or mean ± SD, and *p*‐values <0.05 are considered significant (*p < 0.05; **p < 0.01; ***p < 0.001; ****p < 0.0001; ns, not significant).

## Author Contributions

Varun Sasidharan Nair and Zheng Yu: Formal analysis, investigation, methodology, validation, software, visualization, writing‐original draft. HA: Formal analysis, investigation, visualization. Agnes Bonifacius: Methodology, visualization, writing – review, and editing. Beate Pietzsch: Methodology, validation. Dirk H. Busch, Luka Cicin‐Sain, Fabian Müller, Kilian Schober, Britta Eiz‐Vesper: Methodology, validation, data curation, visualization, resources, writing – review and editing. Stefan Floess: Conceptualization, data curation, formal analysis, methodology, validation, visualization, writing – review and editing. Jochen Huehn: Conceptualization, formal analysis, funding acquisition, investigation, methodology, supervision, validation, visualization, writing – review and editing.

## Funding

The authors have nothing to report.

## Conflicts of Interest

The authors declare no conflicts of interest.

## Peer Review

The peer review history for this article is available at https://doi.org/10.1002/eji.70084.

## Ethics Statement

This research was conducted in accordance with the Declaration of Helsinki and approved by the Ethics Committee of Hannover Medical School (MHH Ethics Committee votes 3639‐2017, 9001_BO‐K, 8147_BO_K_2018, and 9255_BO_K_2020).

## Supporting information




**Supporting Information file 1**: eji70084‐sup‐0001‐Table.xlsx

**Supporting Information file 1**: eji70084‐sup‐0002‐SuppMat.pdf

## Data Availability

RNAseq data generated in this study have been deposited in the NCBI GEO database under accession code GSE291537.
