# Supplementary material for: Epigenetically Controlled ZEB2 Expression Promotes the Cytotoxic Potential of CMV‐Specific CD8+ T Cells
Source: Eur J Immunol. 2025 Nov 6;55(11):e70084. doi: 10.1002/eji.70084 (PMC12590920; doi:10.1002/eji.70084)
Supplement: Supplementary file 2 — Supporting Information file 1: eji70084‐sup‐0002‐SuppMat.pdf [file EJI-55-e70084-s001.pdf]

Supporting Information

Epigenetically controlled *ZEB2* expression promotes the cytotoxic potential of CMV-specific CD8$^+$ T cells

Varun Sasidharan Nair, Zheng Yu, Hosein Ahmadi, Agnes Bonifacius, Beate Pietzsch, Dirk H. Busch, Luka Cicin-Sain, Fabian Müller, Kilian Schober, Britta Eiz-Vesper, Stefan Floess, Jochen Huehn

Supplementary Materials and Methods

Supplementary Figures 1-6

Supplementary Materials and Methods

Antibodies

All antibodies (see Supplementary Material and Methods) were purchased from BioLegend and BD Biosciences. For the isolation of $CD8^+$ T cell subsets, anti-CD3 AF488, anti-CD8 APC, anti-CD45RA PerCP/Cy5.5, anti-CCR7 BV421, anti-CD62L PE/Cy7, anti-CD28 PE-Texas Red, and anti-CD95 PE were used. For the isolation of $CD4^+$ T cell subsets, anti-CD4 APC, anti-CD45RA PerCP/Cy5.5, anti-CCR7 BV421, anti-CD27 BV510, anti-CD28 PE/Cy5, anti-CD57 PE, and anti-CD244 FITC were used. For the isolation of retrovirally-transduced $CD8^+$ T cells, anti-CD8 APC and anti-mouse TCR APC/Cy7 were used.

Isolation of $CD4^+$ T cell subsets

PBMCs were enriched for $CD4^+$ cells using the autoMACS system and either anti-CD4 MicroBeads (positive fraction) or anti-CD8 MicroBeads (negative fraction). The enriched cells were then stained and sorted by flow cytometry into the following T cell subsets: T_N ($CD4^+CD28^+CD45RA^+CCR7^+$), T_{CM} ($CD4^+CD28^+CD45RA^-CCR7^+$), T_{EM} ($CD4^+CD28^+CD45RA^-CCR7^-$), and T_{CD4CTL} ($CD4^+CD57^+CD27^-CD28^-CD244^+$).

In vitro culture of $CD8^+$ T cell subsets

$CD8^+$ T_N and T_{EMRA} cells were cultured *in vitro* as previously described [1]. Briefly, sorted $CD8^+$ T_N and T_{EMRA} cells were stimulated with plate-bound anti-CD3 (OKT3; 1 µg/ml; BioLegend) and anti-CD28 (CD28.2; 0.5 µg/ml; BioLegend) antibodies and cultured in TexMACS medium supplemented with 100 IU/ml human IL-2 at 37°C with 5% CO_2. Every 5 days during the 30-day culture period, cells were harvested, washed and an aliquot taken for pyrosequencing. The remaining cells were restimulated with plate-bound anti-CD3 and anti-CD28 antibodies (both 0.5 µg/ml) and cultured in fresh TexMACS medium supplemented with 100 IU/ml recombinant human (rh) IL-2.

CRISPR/Cas9-mediated *ZEB2* KO

For *ZEB2* KO, we performed RNP transfection as described by Oh et al. [2], making small modifications as recommended by IDT. The following three pre-designed Alt-R® CRISPR-Cas9 crRNAs (IDT) were used to generate RNP complexes:

Hs.Cas9.*ZEB2*.1.AA: 5’-TGACGGTATTGCCAACCCTC-3’

Hs.Cas9.*ZEB2*.1.AB: 5’-TTGGCACGCCAGAGGCCAAT-3’

Hs.Cas9.*ZEB2*.1.AD: 5’-CAAGCGCTTGACATCACTGA-3’

For each crRNA, duplexes were prepared by mixing equimolar amounts of crRNA and tracrRNA (IDT), followed by annealing at 95°C for 5 minutes and cooling to room temperature.

To prepare the RNP complex for each crRNA, 9 µl of crRNA-tracrRNA duplexes were mixed with 3.5 µl of Alt-R™ S.p. Cas9 Nuclease V3 (610 pmol; IDT) and incubated for 20 minutes at room temperature. Prior to transfection, the three RNP complexes were mixed in an equimolar ratio. The non-targeting Alt-R® CRISPR-Cas9 Negative Control crRNA #1 (IDT) was used as a negative control and the RNP complex was prepared as described above. For each transfection, 1 x 10^6 cultured $CD8^+$ effector T cells were resuspended in 100 µl primary cell nucleofection solution (P3 Primary Cell 4D-Nucleofector X kit; Lonza) with the addition of 5 µl RNPs (*ZEB2* targeting or negative control) and 3 µl of electroporation enhancer buffer (Alt-R® Cas9 Electroporation Enhancer; IDT) at room temperature. Electroporation was performed with a 4D Nucleofector (Lonza) using the EH-100 programme as recommended by Oh et al. [2]. Transfected cells were cultured overnight in TexMACS™ medium supplemented with 100 IU/ml rhIL-2 and then stimulated with plate-bound anti-CD3 and anti-CD28 antibodies (both 0.5 µg/ml) for indicated time points in TexMACS™ medium supplemented with 100 IU/ml rhIL-2. After the culture, the cells were harvested and used for subsequent experiments. Notably, the decreased viability occurred specifically in *ZEB2* RNP-transfected cells, while the scrambled control did not show a reduced viability. This suggests that the observed decrease in viability is not due to electroporation itself, given that the same programme and conditions were used across all experiments. For all profiling and functional assays, cells were sorted on day 2 after CRISPR-Cas9-mediated genome editing, before strong differences in viability were observed. Furthermore, only viable cells were sorted and cells were carefully counted to ensure equal numbers were used for the scrambled control and *ZEB2* KO conditions before conducting the functional assays.

RNAseq – processing and analysis

For processing and analysis of RNAseq data, raw reads (.fastq files) were imported into the CLC Genomics Workbench 23.0.4 (Qiagen) as paired reads for further analysis [3]. The quality of the raw reads was checked and adapters were excluded with a quality score limit of 0.05 and maximum number of ambiguities of 2. The trimmed sequences were then mapped to the human genome hg38 reference genome using standard alignment settings: match score 1, mismatch cost 2, gap cost 3, length fraction 0.5 and similarity fraction 0.8. Differential expression analyses were performed using gene expression (GE) tracks generated by the "RNA-Seq Analysis" tool, and data were normalised using the Trimmed Mean of M method [4]. Read counts for each gene were then converted into transcripts per million (TPM) values. To generate a heat map, Euclidean distances were calculated between clusters with filtering based on a minimum FDR *p*-value and fold change of 0.05 and 1.5, respectively. The differentially expressed genes (DEGs) with log2 fold change >1.5 and *p*-value <0.05 cut-offs were subjected to functional annotation analyses based on Gene Ontology Biological Process

enrichment, using the "Database for Annotation, Visualization and Integrated Discovery" (DAVID) [5]. In addition, Z-scores for heat maps were calculated from TPM values, as previously reported [6].

Retroviral transduction

Retroviral transduction of $CD8^+$ T cells with a CMV-specific TCR was performed as previously described [1]. Briefly, the virus-packaging RD114 cells were seeded at 1.5 x 10^6 cells/well in a 6-well plate, 1 day prior to transfection. The following day, pMP71-TCR 5-2 vectors were transfected into RD114 cells using calcium phosphate precipitation. The transfected cells were incubated at 37°C with 5% CO_2 for 15 hours and the medium was then replaced with fresh DMEM. The supernatant containing the retroviral particles was harvested 3 days later. For the retroviral transduction, $CD8^+$ T_{EM} and T_{EMRA} cells from CMV-seronegative HLA-A*02:01$^-$ healthy individuals were sorted together and stimulated with plate-bound anti-CD3 (1 µg/ml) and anti-CD28 (0.5 µg/ml) for 48 hours. Retroviral transduction was performed by spinoculation. Five days later, successfully transduced $CD8^+$mTCR$^+$ T cells were sorted by flow cytometry and used for *ZEB2* KO. Prior to *ZEB2* KO, sorted cells were washed and rested overnight in TexMACS^{TM} medium supplemented with 100 IU/ml rhIL-2. Cells were harvested and restimulated for 72 hours with plate-bound anti-CD3 and anti-CD28 (both 0.5 µg/ml) in TexMACS^{TM} medium supplemented with 100 IU/ml rhIL-2, followed by CRISPR/Cas9-mediated *ZEB2* KO as described above. At the end of the culture, cells were harvested and used for the *in vitro* cytotoxicity assay.

References Supplementary Materials and Methods

1 **Yu, Z., Sasidharan-Nair, V., Buchta, T., Bonifacius, A., Khan, F., Pietzsch, B., Ahmadi, H., Beckstette, M., Niemz, J., Hilgendorf, P., Mausberg, P., Keller, A., Falk, C., Busch, D. H., Schober, K., Cicin-Sain, L., Müller, F., Brinkmann, M. M., Eiz-Vesper, B., Floess, S. and Huehn, J.,** DNA methylation profiling identifies TBKBP1 as potent amplifier of cytotoxic activity in CMV-specific human $CD8^+$ T cells. *PLoS Pathog* 2024. **20**: e1012581.

2 **Oh, S. A., Seki, A. and Rutz, S.,** Ribonucleoprotein transfection for CRISPR/Cas9-mediated gene knockout in primary T cells. *Curr Protoc Immunol* 2019. **124**: e69.

3 **Liu, C. H. and Di, Y. P.,** Analysis of RNA sequencing data using CLC Genomics workbench. *Methods Mol Biol* 2020. **2102**: 61-113.

4 **Robinson, M. D. and Oshlack, A.,** A scaling normalization method for differential expression analysis of RNA-seq data. *Genome Biol* 2010. **11**: R25.

5 **Huang, D. W., Sherman, B. T., Tan, Q., Collins, J. R., Alvord, W. G., Roayaei, J., Stephens, R., Baseler, M. W., Lane, H. C. and Lempicki, R. A.,** The DAVID Gene Functional Classification Tool: a novel biological module-centric algorithm to functionally analyze large gene lists. *Genome Biol* 2007. **8**: R183.

6 **Malone, B. M., Tan, F., Bridges, S. M. and Peng, Z.,** Comparison of four ChIP-Seq analytical algorithms using rice endosperm H3K27 trimethylation profiling data. *PLoS One* 2011. **6**: e25260.

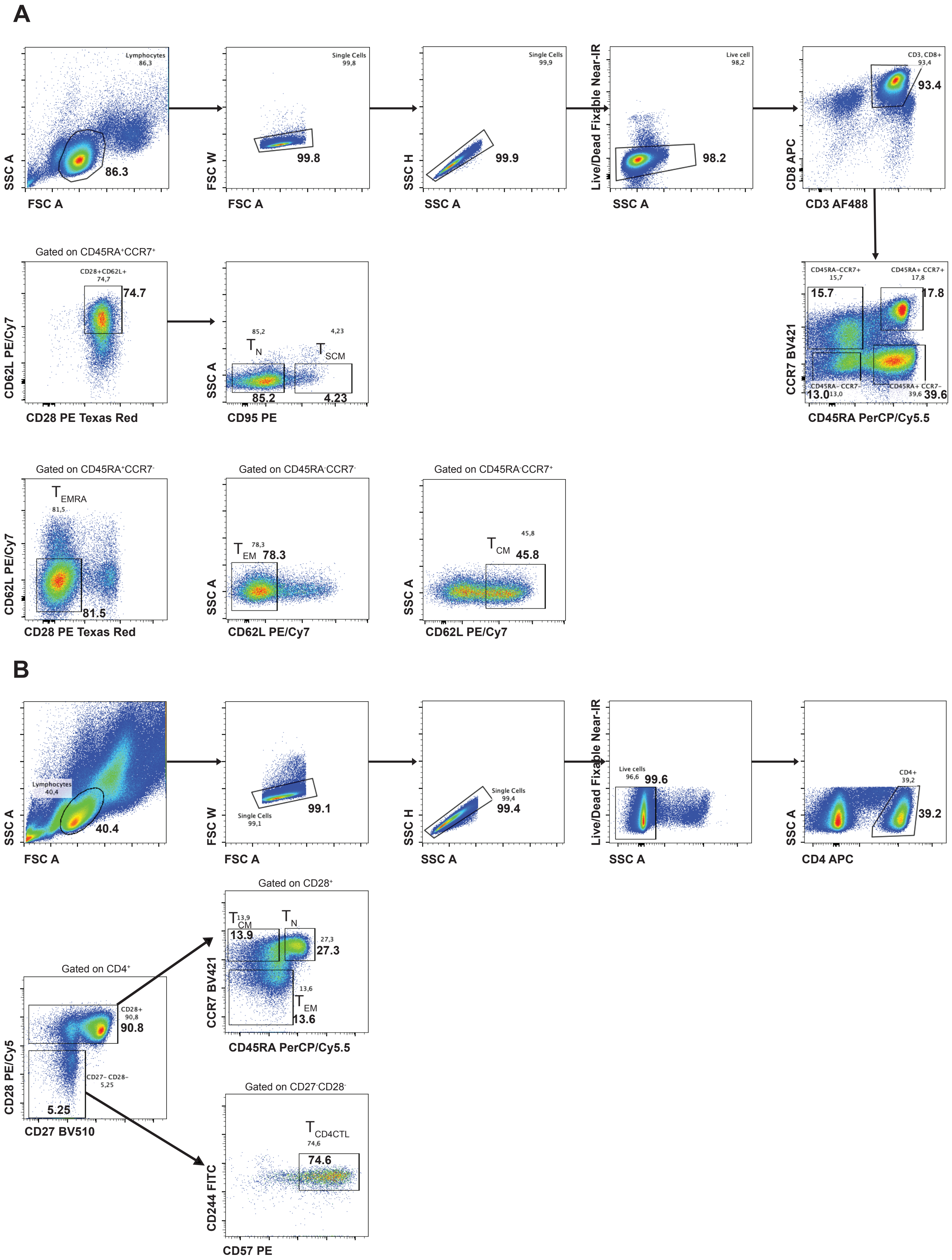

Supplementary Figure 1: Sorting strategy of $CD8^+$ and $CD4^+$ T cell subsets. $CD8^+$ and $CD4^+$ T cell subsets from CMV seropositive healthy donors were sorted by flow cytometry and RNA and gDNA were isolated and subjected to qRT-PCR and pyrosequencing, respectively. Representative flow cytometric plots show the gating strategy for the sorting of (A) $CD8^+$ T_N, T_{SCM}, T_{CM}, T_{EM} and T_{EMRA} cells, and (B) $CD4^+$ T_N, T_{CM}, T_{EM}, and T_{CD4CTL} cells.

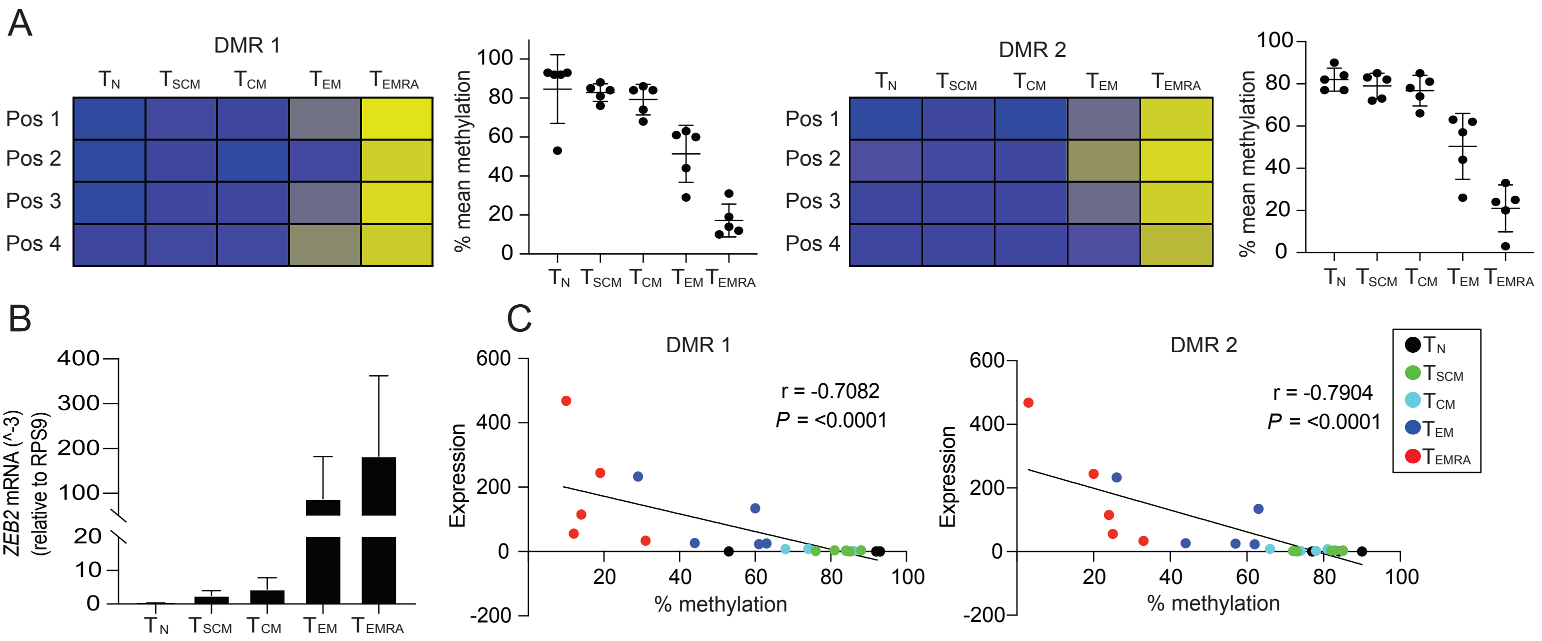

Supplementary Figure 2: Methylation status of *ZEB2* DMRs correlates with *ZEB2* expression in CD8⁺ T cell subsets from CMV-seronegative donors. CD8⁺ T subsets from CMV-seronegative healthy donors were sorted by flow cytometry and gDNA was isolated and subsequently treated with sodium bisulfite. The bisulfite-converted DNA was subjected to pyrosequencing using primers targeting *ZEB2* DMRs to access the methylation pattern. Meanwhile, RNA was also isolated from sorted CD8⁺ T cell subsets to measure *ZEB2* mRNA levels in each subset. (A) Methylation profiles of two defined *ZEB2* DMRs in human CD8⁺ T cell subsets (T_N, T_{SCM}, T_{CM}, T_{EM} and T_{EMRA} cells) from one representative donor (left) and mean methylation levels from five independent donors (right). Methylation values of individual CpG motifs have been transformed into a colour-coded box, ranging from yellow (0%) to blue (100%) (left). (B) Bar plots show the transcriptomic expression of *ZEB2* relative to the housekeeping gene *RPS9* in CD8⁺ T cells from CMV-seropositive donors (n=5). (C) Scatterplots show the correlation between mean methylation levels of *ZEB2* DMR1 (left) and DMR2 (right) with *ZEB2* expression in CD8⁺ T_N (black), T_{SCM} (green), T_{CM} (cyan), T_{EM} (blue) and T_{EMRA} (red) cells from five independent donors. Linear regression analysis was performed to determine the correlation.

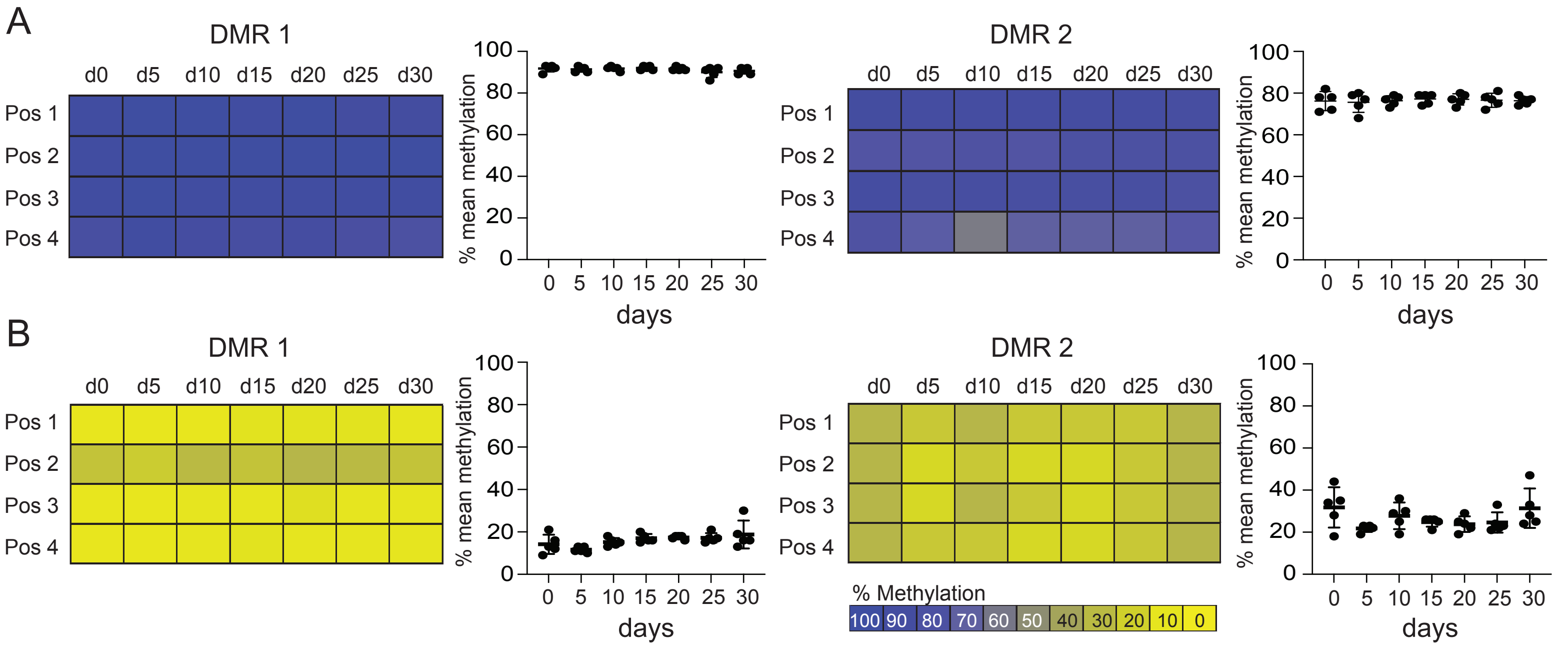

Supplementary Figure 3: *In vitro* cultured CD8⁺ T_N and T_{EMRA} cells show stable methylation status of *ZEB2* DMRs. CD8⁺ T cell subsets (T_N and T_{EMRA}) were sorted and cultured under anti-CD3/CD28 stimulation in presence of IL-2 for 30 days. Cells were harvested every 5 days, washed, and an aliquot was used for pyrosequencing to determine changes in methylation levels of *ZEB2* DMRs. Methylation values of individual CpG motifs in indicated *ZEB2* DMRs from CD8⁺ (A) T_N and (B) T_{EMRA} subsets were plotted in a heat map (left) and mean methylation levels for all CpG motifs within the indicated DMRs from 5 independent donors (mean±SD) are shown in the graphs (right). Methylation values of individual CpG motifs have been transformed into a colour-coded box, ranging from yellow (0%) to blue (100%).

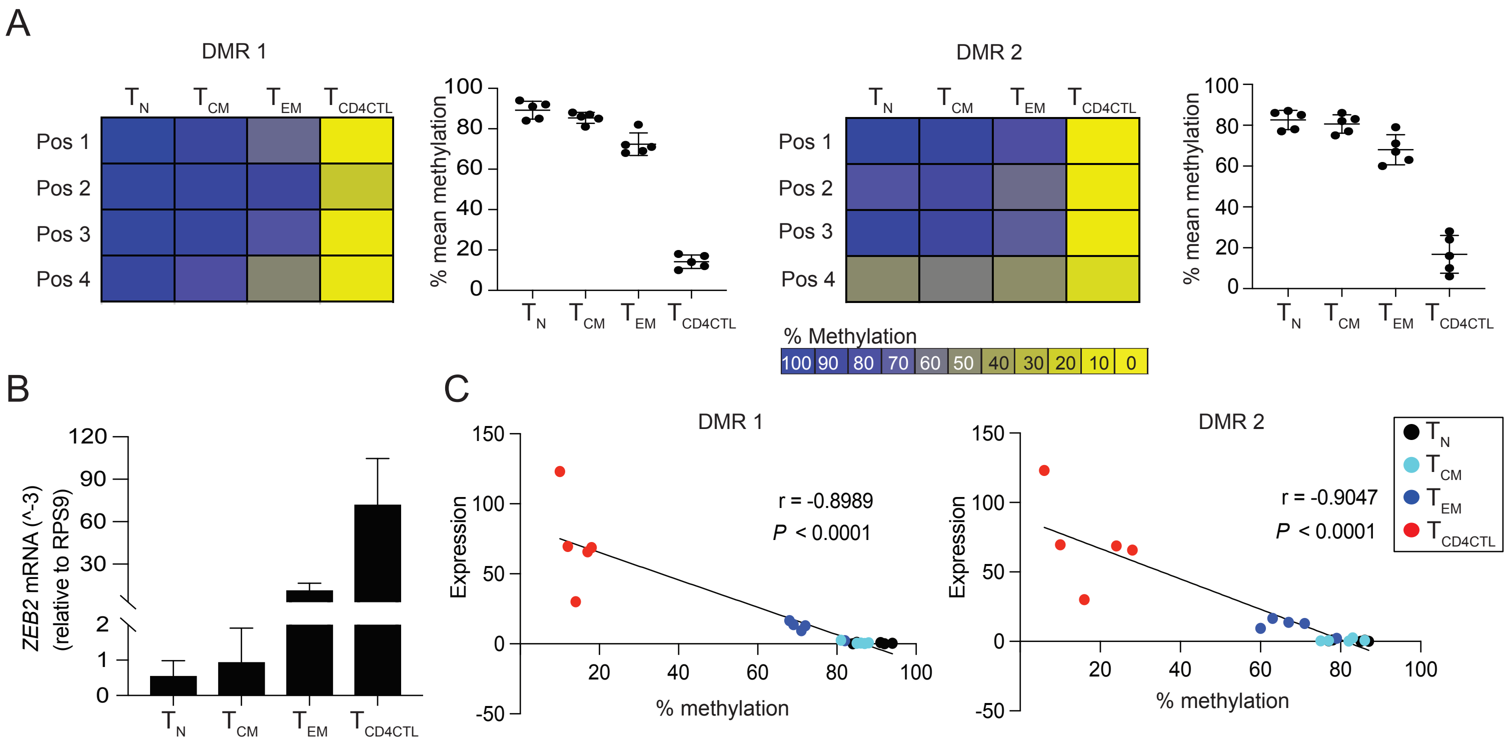

Supplementary Figure 4: Cytotoxic CD4⁺ T cells show pronounced demethylation at *ZEB2* DMRs and increased *ZEB2* expression. CD4⁺ T subsets from CMV-seropositive healthy donors were sorted by flow cytometry and gDNA was isolated and subjected to pyrosequencing, as described previously. (A) Methylation profiles of two defined *ZEB2* DMRs in human CD4⁺ T cell subsets (T_N, T_{CM}, T_{EM} and T_{CD4CTL} cells) from one representative (left) and mean methylation levels from 5 independent donors (right). (B) Bar plots show the transcriptomic expression of *ZEB2* relative to the housekeeping gene *RPS9* in CD4⁺ T cells from seropositive donors (n=5). (C) Correlation between methylation level of *ZEB2* DMRs and transcriptomic expression of *ZEB2*. Scatterplot shows mean methylation of *ZEB2* DMR1 (left) and DMR2 (right) correlated with *ZEB2* expression in a population of T_N (black), T_{CM} (cyan), T_{EM} (blue) and T_{CD4CTL} (red) cells (n=5).

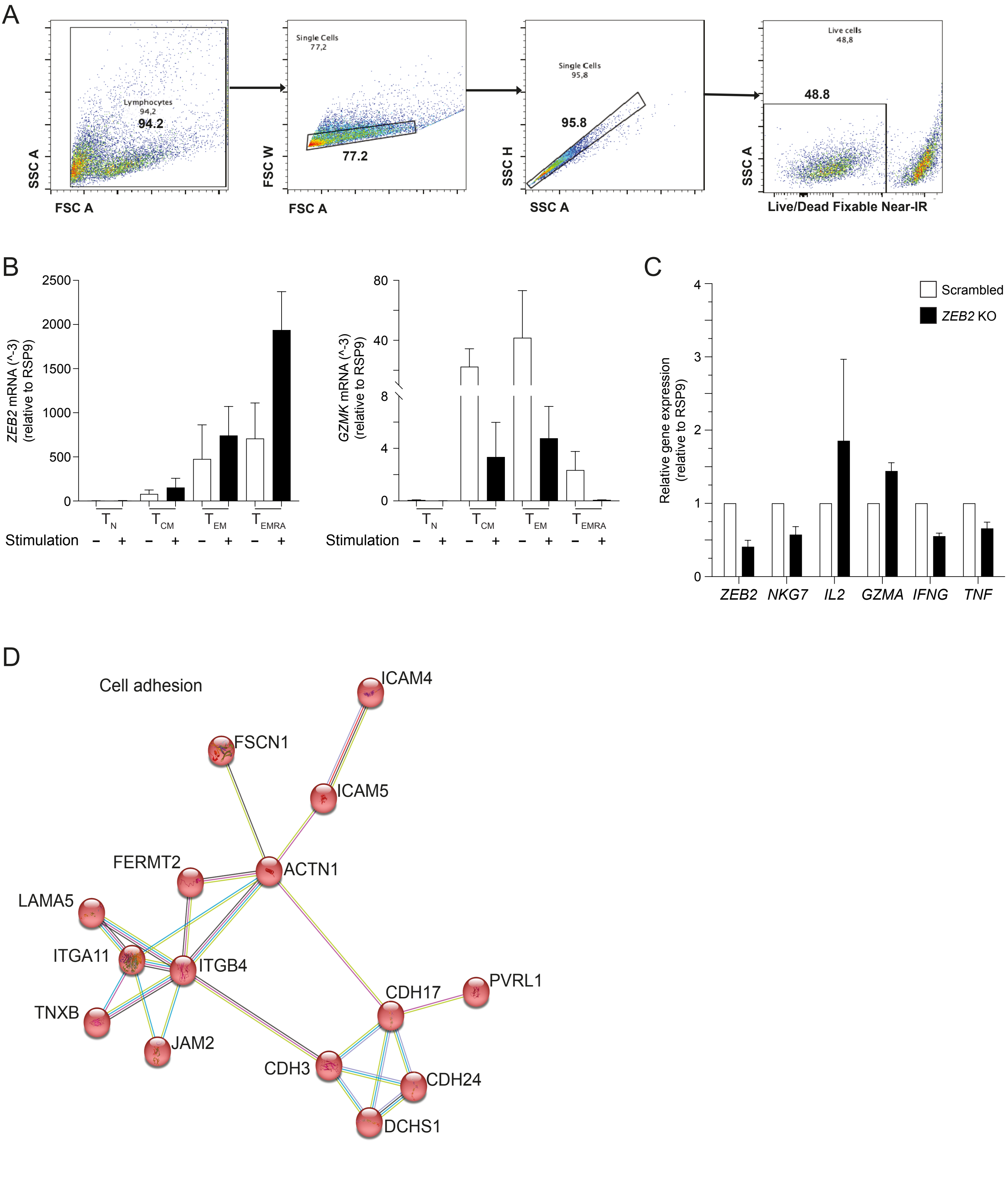

Supplementary Figure 5: Characterisation of CD4⁺ and CD8⁺ T cells in the presence or absence of *ZEB2*. (A) CD8⁺ T_{EM} and T_{EMRA} cells were isolated from CMV-seronegative donors and *ZEB2* was deleted using CRISPR/Cas9-mediated editing. Edited cells were subjected to RNA-sequencing and functional validation by cytotoxic killing assay. Representative flow cytometric plots showing the gating strategy to assess T cell viability after *ZEB2* KO. After nucleofection, CD8⁺ T cells were stained with LIVE/DEAD® Fixable Near-IR Dead Cell Dye, followed by flow cytometric analysis. (B) Bar plots show the transcriptomic expression of *ZEB2* (left) or *GZMK* (right) relative to the housekeeping gene *RPS9* in non-stimulated (white bars) or stimulated (black bars) CD8⁺ T cell subsets from CMV-seropositive donors (n=4-6). (C) T_{CD4CTL} were isolated from CMV-seropositive donors and *ZEB2* was deleted using CRISPR/Cas9-mediated editing. Two days after nucleofection, the *ZEB2* KO efficacy as well as expression of *NGK7, IL2, GZMA, IFNG* and *TNF* was assessed by qRT-PCR. Bar plot shows target gene expression relative to *RPS9* expression in *ZEB2* KO (black bars) and scrambled control (white bars) samples (n=3). (D) Protein-protein interaction network of cell adhesion-related genes from downregulated DEGs in *ZEB2* KO using the STRING database. The functional association was represented by the colour saturation of the edges.

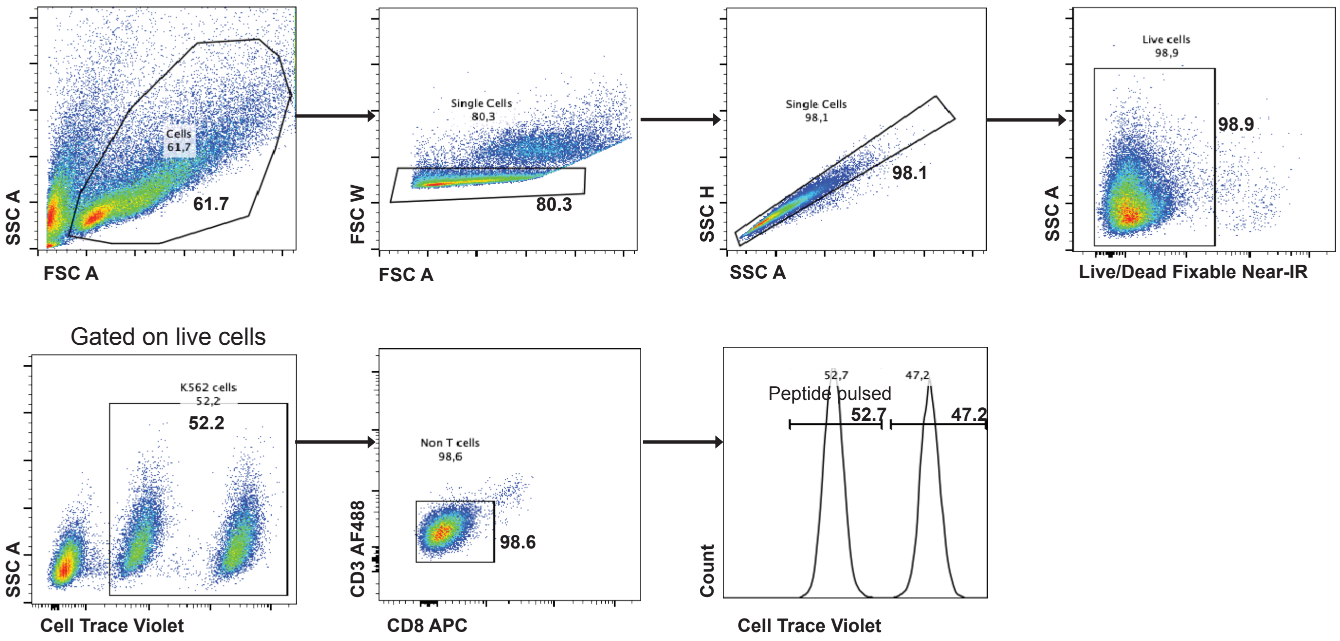

Supplementary Figure 6: Analysis of *ZEB2* KO CD8^{+} T cells in the cytotoxic killing assay. Gating strategy to identify the target cells (CMVpp65 pulsed K562 cells: low concentration of CTV; unpulsed K562 cells: high concentration of CTV) in the cytotoxic killing assay. Target cells were analyzed after gating on CD3^{-}CD8^{-} non-T cells. The killing efficacy of *ZEB2* KO and control T cells was determined by comparing the frequency of peptide-pulsed versus unpulsed cells.
